# Characteristics and Prognostic Significance of Preoperative Magnetic Resonance Imaging-Assessed Circumferential Margin in Rectal Cancer

**DOI:** 10.1155/2015/410150

**Published:** 2015-05-19

**Authors:** Xiaoji Ma, Xinxiang Li, Linghui Xu, Debing Shi, Tong Tong, Dan Huang, Ying Ding, Sanjun Cai, Junjie Peng

**Affiliations:** ^1^Department of Colorectal Surgery, Fudan University Shanghai Cancer Center, Shanghai 200032, China; ^2^Department of Oncology, Shanghai Medical College, Fudan University, Shanghai 200032, China; ^3^Department of Radiology, Fudan University Shanghai Cancer Center, Shanghai 200032, China; ^4^Department of Pathology, Fudan University Shanghai Cancer Center, Shanghai 200032, China; ^5^Department of Biostatistics, University of Pittsburgh, Pittsburgh, PA, USA

## Abstract

*Purpose*. To study the characteristics and prognostic significance of preoperative magnetic resonance imaging- (MRI-) assessed circumferential margin (CRM) in rectal cancer. *Methods*. Patients underwent preoperative high resolution pelvic MRI, followed by resection of primary tumor. The relationship between MRI-assessed CRM and pathological CRM (pCRM) was studied, and survival analysis was used to determine the prognostic significance of MRI-assessed CRM. *Results*. Of all the 203 patients, the total accuracy of MRI-assessed CRM for predicting involvement of pCRM was 84.2%, sensitivity was 50%, and specificity was 86.8%. Anterior tumors were more possible to assess involvement of CRM by MRI, while the false positive rate was significantly higher than lateral or posterior tumor (87.5% versus 50%, *p* = 0.0002). The 3-year local recurrence, disease-free survival, and overall survival rates were 35.6%, 58.1%, and 85.2% in patients with involved mrCRM, compared with 8.9%, 78.9%, and 92.3% in patients with clear mrCRM. In multivariate analysis, MRI-assessed CRM found an independent risk factor for local recurrence, with a hazard ratio of 3.49 (*p* = 0.003). *Conclusions*. High resolution MRI was accurate to assess CRM preoperatively, while anterior tumor should be assessed more cautiously. Involvement of mrCRM was significantly associated with local recurrence regardless of pCRM status.

## 1. Background

Colorectal cancer is the third most common malignancy in China, and the incidence and mortality rates are continuing to rise in recent years. Although the proportion of colon cancer is increasing, rectal cancers still account for over 50% of all colorectal cancers in China.

Management of rectal cancer is particularly challenging for colorectal specialists, as distant metastasis, local recurrence, and quality of life, especially anal sphincter preservation, are almost of the same importance in a successful treatment. In the era of multidisciplinary treatment, for locally advanced rectal cancer, preoperative chemoradiation followed by total mesorectal excision has been proved to obtain improved local recurrence, reduced acute and chronic toxicity, and improved sphincter preservation rates [1]. However, several new controversies had emerged such as the accuracy of preoperative staging, the surgical distal margin after preoperative chemoradiation, and pathological assessment of tumor regression and surgical margin.

Circumferential margin (CRM) is one of the most important parameters assessed by preoperative imaging and postoperative pathology. Most studies have confirmed that involvement of pathological circumferential margin (pCRM) was significantly related to higher rate of local failure and lower disease-free survival time [2–4]. Preoperative high resolution magnetic resonance imaging (MRI) has been studied to predict pathological involvement of CRM in MERCURY study [5]. Preoperative MRI assessment of CRM (mrCRM) was found to have higher accuracy than digital examination. However, the detailed feature of CRM involvement in MRI imaging, such as location, tumor spectrum, and its relationship to clinical characteristics, is still unknown. Meanwhile, the prognostic significance of mrCRM still needs to be further studied.

Our study was designed to study the relationship between the mrCRM and other radiological or pathological characteristics and assess the prognostic significance of mrCRM. To clarify the natural characteristics of mrCRM, our study retrospectively selected patients with rectal cancer, who received preoperative high resolution MRI scanning and underwent surgical resection as the primary treatment.

## 2. Materials and Methods

### 2.1. Ethics

The study was conducted at the Fudan University Shanghai Cancer Center. This study was approved by the Fudan University Shanghai Cancer Center Institutional Ethics Committee. According to hospital routine, patients are asked to provide a written informed consent after their admission that their clinical and outcome information will be used in future scientific studies. Patients' records were anonymized and deidentified prior to analysis.

### 2.2. Patients

From the prospectively collected database of colorectal cancer in Fudan University Shanghai Cancer Center, all patients with rectal cancer, operated with curative intent in our hospital between January 2007 and June 2013, were screened for this study. The rectal cancer is defined as within 12 cm from anal verge by preoperative colonoscopy. As preoperative treatment may alter the status of pathological CRM, all patients undergoing preoperative treatment were not included in current study. Other exclusion criteria included unresectable primary tumors, no preoperative high resolution pelvic MRI, missingness of pathologically assessed CRM status, metastatic disease, local excision, and history of other malignancies.

A total of 203 patients were finally collected to have complete information of histopathological and radiological information. Pathological CRM was assessed for all patients. This study was approved by the Fudan University Shanghai Cancer Center Institutional Ethics Committee.

### 2.3. Follow-Up

The adjuvant treatment of patients was followed by doctors' guidance and recorded in our database. The patients were followed up according to institutional protocol. Briefly, clinical assessment and CEA test were required every 3 months in the first 2 years after surgery and every 6 months thereafter until the fifth year. Chest CT and abdominopelvic CT or MRI scans were required every 6 months in the first 2 years after surgery and annually until the fifth year. Local recurrence or distant metastases were confirmed by progressive enlargement of lesions in radiological images, and biopsy was performed if clinically indicated. Between November 2013 and January 2014, all surviving patients received an additional follow-up by mail or telephone for the purpose of current study.

### 2.4. Surgery and Pathological Assessment

All patients in current study underwent radical resection with the principle of total mesorectal excision for middle and low rectal cancer, and partial mesorectal excision, a minimum of 5 cm beyond the distal edge of primary tumor, was performed for high rectal cancer. The pathological assessment of resected specimens was performed according to the College of American Pathologists Consensus Statement [6]. A clear pathological CRM (pCRM) is defined as >1 mm distance from the tumor to surgical margin [7, 8]. Each rectal cancer was staged according to the AJCC Cancer Staging Manual [9].

### 2.5. MRI Assessment

**Figure 1:**
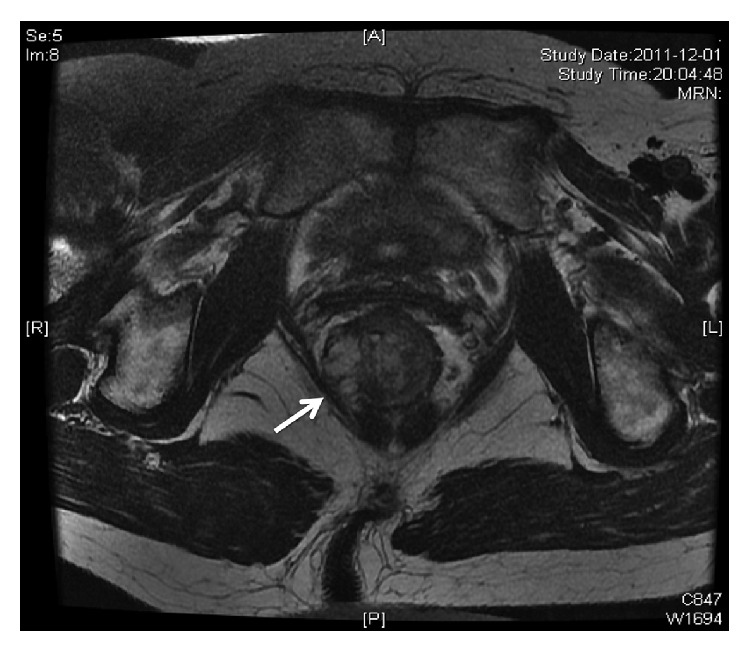
T2-weighted axial thin section magnetic resonance imaging scan. This scan shows a low rectal tumor with involvement of the potential circumferential resection margin (white arrow).

The high resolution scan parameters in the preoperative MRI were used for each patient according to the similar protocol of MERCURY study [5]. The T2-weighted fast spin echo sequence with a thin 2 mm section was mainly used for preoperative assessment. Images are made in the sagittal, coronal, and axial plane. The cranial border of the field of view is L5 and the caudal border is below the anal canal. No intrarectal coli or contrast was used. Intravenous gadolinium-enhanced scanning was routinely used for each patient, but its imaging was not assessed in this study. A clear MRI-assessed CRM (mrCRM) is defined as >1 mm distance from the tumor to mesorectal fascia in high and middle rectal tumors or >1 mm distance from the tumor to levator muscle in low rectal tumors. The distance of closest mrCRM for each case in axial plane of MRI images was measured and recorded (Figure 1).

Specifically, the scope of primary tumor and the location of closest mrCRM in the bowel wall circumference (anterior, lateral, and posterior) were recorded in axial plane of MRI images. The relationship between primary and peritoneal reflex was also recorded as above or below peritoneal reflex in sagittal plane of MRI images.

### 2.6. Statistics

Local recurrence (LR) time was calculated from surgery to the time when cancer recurrence in the surgical bed or pelvis was discovered on physical examination, colonoscopy, or imaging studies. Disease-free survival (DFS) time was defined as the time from surgery to the time of locoregional or distant recurrence. Overall survival (OS) time was defined from surgery until death as a result of any cause.

The rates of LR, DFS, and OS were computed using the Kaplan-Meier method. Log-rank tests were performed to compare differences among survival curves in univariate analyses. Logistic regression was performed to identify preoperative clinical characteristics of patients with MRI-assessed involvement of CRM, and odds ratios with 95% of confidence interval were also calculated.

The associations between mrCRM and pCRM or other characteristics were assessed by Pearson's chi-square test in crosstab tables, and the accuracy, sensitivity, specificity, positive predictive value (PPV), and negative predictive value (NPV) were calculated. Independent* t*-test was used to compare the differences of the value of distance from tumor to the closest mesorectal fascia. The Cox regression model was performed in the univariate and multivariate analyses for the DFS time and the OS time, and hazard ratios were estimated with corresponding 95% confidence intervals (95% CI). A *p* value of < 0.05 was considered statistically significant.

## 3. Results

### 3.1. Patient Characteristics

Of all the 203 patients, the median age was 61 years, ranging from 27 to 84 years. The baseline characteristics and the correlations between mrCRC and clinical/radiological characteristics were presented in Table 1. MRI-assessed involvement of CRM was found to be associated with mrT and mrN classification, circumferential tumor location, distance from anal verge, AJCC TNM stage, and pCRM (Table 1).

To predict the involvement of mrCRM, preoperative available variables were selected in the multivariate analysis, including age, sex, circumferential tumor location, tumor distance from anal verge, preoperative CEA, tumor grade, and MRI-assessed T and N classification. The multivariate analysis showed that MRI-assessed N classification, circumferential tumor location, and tumor distance from anal verge were associated with the involvement of mrCRM. The odds ratios were 2.41 (95% CI, 1.04–5.58, *p* = 0.039) in patients with mrN(+) versus mrN(−), 3.15 (95% CI, 1.27–7.77, *p* = 0.013) in patients with anterior tumors versus lateral/posterior tumors, and 2.83 (95% CI, 1.12–7.15, *p* = 0.028) in patients with tumors ≤5 cm from anal verge versus >5 cm from anal verge.

### 3.2. Interaction between mrCRM and pCRM or Circumferential Tumor Location

Of all the 203 patients, 32 patients (15.8%) found involvement of mrCRM, while 14 patients (6.9%) found involvement of pCRM. The total accuracy of mrCRM for predicting involvement of pCRM was 84.2%, with sensitivity 50%, specificity 86.8%, the NPV 95.9%, and the PPV 21.9%.

In our series, the circumferential locations of closest mrCRM in bowel wall were anterior (107 patients, 52.7%), lateral (84 patients, 41.4%), and posterior (12 patients, 5.9%). Tumors located laterally and posteriorly were categorized into one group. We found a significant higher rate of involved CRM by MRI assessment in anterior tumors as compared to in lateral/posterior tumors (22.4% versus 8.3%, *p* = 0.023). However, involvement of pCRM was similar between anterior tumors and lateral/posterior tumors (6.5% versus 7.3%, *p* = 0.83). For anterior tumors, only 3 of 24 patients (12.5%) with involvement of mrCRM were pathologically confirmed by pCRM, while in lateral or posterior tumors, 4 of 8 patients (50%) with involvement of mrCRM were pathologically confirmed by pCRM (*p* = 0.0002).

### 3.3. Survival Analyses and Prognostic Significance of mrCRM

Of all the 203 patients, the median follow-up of the surviving patients was 30 months (range, 6–75 months). 24 patients (11.8%) died of cancer or other reasons, 27 patients (13.3%) had local recurrence, and 155 patients (75.9%) were still free of disease at last follow-up. The 3-year LR, DFS, and OS rates were 14.7%, 74.3%, and 90.4%, and the 5-year OS rate was 76.9%.

**Figure 2:**
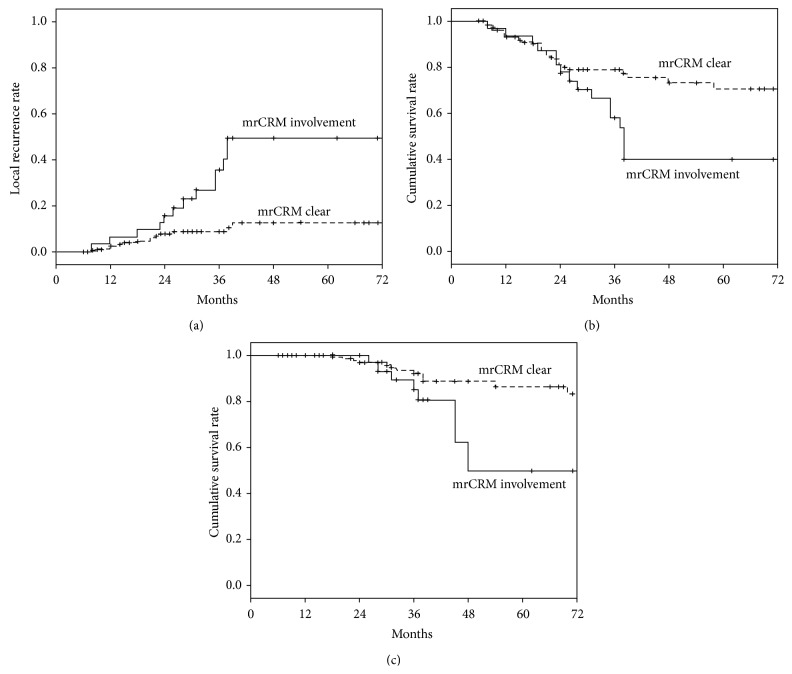
Kaplan-Meier survival plots for time to local recurrence, disease-free survival, and overall survival by circumferential margin status: (a) plot of local recurrence; (b) plot of disease-free survival; (c) plot of overall survival. mrCRM, magnetic resonance imaging-assessed circumferential resection margin.

**Figure 3:**
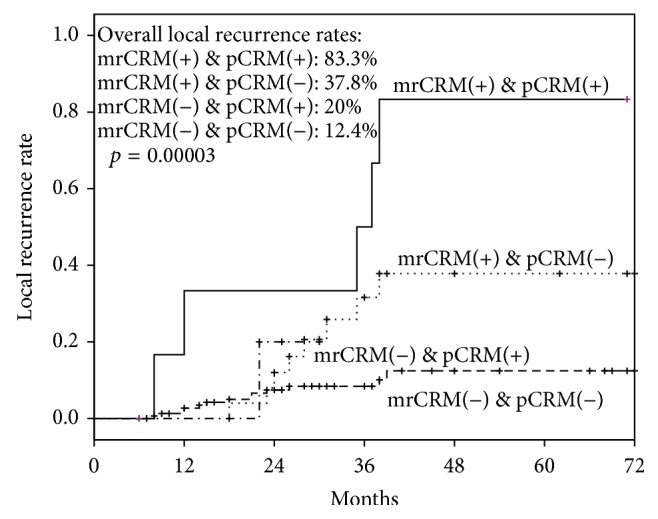
Kaplan-Meier survival plots for time to local recurrence in patients in groups of mrCRM(+) & pCRM(+), mrCRM(+) & pCRM(−), mrCRM(−) & pCRM(+), and mrCRM(−) & pCRM(−).

The association between each radiological/clinicopathological characteristic and patients' outcomes (LR, DFS and OS) was studied by the univariate analysis and was summarized in Table 2. From the univariate analysis, mrCRM was found to be associated with LR, DFS, and OS. The 3-year LR, DFS, and OS rates were 35.6%, 58.1%, and 85.2% in patients with involved mrCRM, compared with 8.9%, 78.9%, and 92.3% in patients with clear mrCRM (Figure 2). The LR rates were calculated in patients with different mrCRM and pCRM status. The overall LR rate was 12.4% in patients with mrCRM-clear and pCRM-clear tumors, while the overall LR rate was 83.3% in patients with mrCRM-involved and pCRM-involved tumors (Figure 3). In 189 patients with clear pCRM, the 3-year LR rate was 8.4% in patients with clear mrCRM, compared with 31.6% in patients with involved mrCRM (*p* = 0.007).

All variables in the univariate analysis were included in the multivariate analysis. From the multivariate analysis for each outcome (LR, DFS, and OS), mrCRM found an independent prognostic factor for LR, with a hazard ratio of 3.49 (95% CI 1.53–7.95, *p* = 0.003). Other factors associated with LR were preoperative CEA and pN classification, with hazard ratios of 2.94 (95% CI 1.29–6.68, *p* = 0.01) and 3.03 (95% CI 1.03–8.92, *p* = 0.045), respectively. Only pN classification was found associated with DFS with a hazard ratio of 3.53 (95% CI 1.64–7.61, *p* = 0.001). Age and pT classification were found associated with OS, with hazard ratios of 2.67 (95% CI 1.10–6.48, *p* = 0.03) and 9.87 (95% CI 1.31–74.1, *p* = 0.026).

## 4. Discussion

In our study, to present the original characteristics of mrCRM and its relationship with pCRM or outcomes, we specifically selected patients with rectal cancer who underwent curative resection of primary tumor as the primary treatment after preoperative MRI scanning. Similar to previous study, our study also found mrCRM was accurate to predict pCRM and involved mrCRM was related to poor outcomes, especially LR rate. Specifically, our study further confirmed that anterior tumors were more prominent to be assessed mrCRM involvement, which was still controversial in previous studies.

Currently, preoperative chemoradiotherapy is the standard treatment for locally advanced rectal cancer to reduce local recurrence. The patient selection criterion is mainly based on preoperative assessment of AJCC TNM stage, which manifested the invasion of primary tumor and metastases in the draining lymphatic pathway. However, inadequate surgical margins and residual tumor cells in the circumferential margin are associated with a high risk of local recurrence, which may be reduced by preoperative treatment [3, 10, 11]. Accurate assessment of preoperative circumferential margin may largely decrease the rate of involvement of pCRM.

Preoperative MRI provides an accurate assessment of the local spread within the pelvis and is crucial for multidisciplinary treatment in rectal cancer. Although preoperative MRI is thought to be the current best method available for predicting the surgically clear circumferential resection margin, there is still insufficient evidence to predict an involved pCRM by a positive mrCRM. Previous studies found inconsistent correlations for CRM between MRI and postoperative pathology. The sensitivity ranged widely from 29% to 100%. The specificity and negative predictive value was high, but the positive predictive value was generally low, ranging from 20% to 89% [12–18]. The European multicenter study MERCURY demonstrated that mrCRM, assessed by preoperative high resolution MRI, gave an accurate prediction of the pCRM [5]. The result was found high in accuracy (87%), specificity (91%), and negative predictive value (94%), but low in sensitivity (64%) and positive predictive value (53%) [19]. Meanwhile, the predicting accuracy was found decreased from 91% in the primary surgery group to 77% in the preoperative treatment group [5]. This discrepancy may be related to the posttreatment scarring by the downsizing of the primary tumor, which may cause difficulty in interpretation of the scans. In our series, mrCRM was also found to have a good accuracy (84.2%) and negative predictive value (95.9%) in predicting pCRM, while the positive predictive value was also much lower.

Moreover, due to the different thickness of mesorectal fascia within in the rectum, the predicting accuracy may be varied with different circumferential tumor location. Kim et al. reported that the accuracy varied from 59% to 96% in patients with anterior, lateral, and posterior tumors [12]. The accuracy was lower in anterior tumors due to the thickness of the mesorectal component that is relatively scant in the anterior rectum. In our series, we found that the rate of mrCRM involvement was found significantly higher for anterior tumors, while pCRM positive rates were similar between anterior tumor and lateral/posterior tumors. Several reasons may attribute to this: although the resection margin was closer for anterior tumors, the surrounding organs, such as seminal vesicle, posterior wall of vagina or prostate, may help surgeons dissect in the right plane. Otherwise, with the guidance of preoperative MRI, compared to lateral/posterior tumors, more extended resections were more possible to be performed for anterior tumors to obtain a clear pCRM, such as resection of seminal vesicle, the posterior wall of vagina or prostate.

Although a positive mrCRM cannot definitively predict an involved pCRM, involvement of CRM in MRI may still have its prognostic significance [20]. In one study, preoperative MRI features were used to select a good prognosis group of patients with rectal cancer to undergo the primary surgery only. MRI-assessed CRM was one of the most important parameters, while lymph node metastasis was not included as a prognostic factor. In 122 patients with good prognosis, the 5-year LR rate was only 3.3%, and only one of the 58 patients with T3N0-2 disease had local recurrence [21]. Using MRI-assessed CRM and lymph node as risk stratification to choose the preoperative treatment, Engelen et al. also reported 2.2% of LR at 3 years [22]. The MERCURY study also proved that MRI-involved CRM was the only preoperative staging parameter that remained significant for OS, DFS, and LR in multivariate analysis. Specifically, the LR rates were 20% and 27% in patients with involved mrCRM and involved pCRM, compared with 7% LR in patients with clear mrCRM or clear pCRM [19].

In our series, we also found a significantly higher LR, DFS, and OS rate in patients with involved mrCRM, and mrCRM was an independent prognostic factor for LR in multivariate analysis. Furthermore, in cases with clear pCRM, significantly higher LR was also observed in patients with involved mrCRM. Therefore MRI-assessed CRM should be considered an important factor in the perioperative treatment selection and in the outcome assessment. The highest LR rate was found in patients with mrCRM-involved and pCRM-involved tumors. Although pCRM was not associated with survival outcomes in our series, this may be attributed to the small number of patients with involved pCRM. However, the interpretation should be careful due to our small number of size in patients with involved pCRM.

In MERCURY study, 41.2% of patients underwent neoadjuvant radiotherapy or chemoradiotherapy, and in our study, 66.1% of patients underwent adjuvant chemotherapy or chemoradiotherapy. MRI-assessed CRM in both studies was proved to be an independent prognostic factor regardless of perioperative treatment. This suggested that more intensified perioperative treatment might be needed in patients with involved mrCRM. For instance, in ACCORD 12/0405 trial, which compared neoadjuvant radiotherapy 45 Gy plus capecitabine with radiotherapy 50 Gy plus capecitabine and oxaliplatin (Capox) in locally advanced rectal cancer, the rate of positive circumferential rectal margins was significantly lower in patients treated with radiotherapy 50 Gy plus Capox [23]. Randomized studies are warranted to confirm the outcomes of patients with involved mrCRM.

## 5. Conclusions

Preoperative mrCRM was accurate to predict involvement of pCRM. Anterior tumor was more possible to be assessed as involved CRM by MRI. Involvement of mrCRM was significantly associated with local recurrence both in patients with or without involvement of pCRM. Neoadjuvant treatment with more intensified therapeutic regimens may be needed in patients with involved mrCRM to improve patients' outcomes.

## Figures and Tables

**Table 1:** The relationship between mrCRM status and clinicopathological characteristics.

	Frequency		mrCRM status				*p* value
			Clear mrCRM		Involved mrCRM		
	Number	%	Number	%	Number	%	
Total	—	—	171	84.2	32	15.8	
Age, years							
<65	113	55.7	94	83.2	19	16.8	0.645
≥65	90	44.3	77	85.6	13	14.4	
Sex							
Male	104	51.2	87	83.7	17	16.3	0.815
Female	99	48.8	84	84.8	15	15.2	
Preoperative CEA							
<5 ng/*μ*L	147	72.4	124	84.4	23	15.6	0.941
≥5 ng/*μ*L	56	27.6	47	83.9	9	16.1	
mrT classification							
mrT1-2	30	14.8	30	100	0	0	0.01
mrT3	173	85.2	141	81.5	32	18.5	
mrN classification							
mrN(−)	114	56.2	102	89.5	12	10.5	0.02
mrN(+)	89	43.8	69	77.5	20	22.5	
Location of closest mrCRM in bowel wall							
Anterior	107	52.7	83	77.6	24	22.4	0.023
Lateral	84	41.4	77	91.7	7	8.3	
Posterior	12	5.9	11	91.7	1	8.3	
Peritoneal reflex relationship							
Above	39	19.2	35	89.7	4	10.3	0.294
Below	164	80.8	136	82.9	28	17.1	
Tumor distance from anal verge							
>5 cm	85	41.9	78	91.8	7	8.2	0.012
≤5 cm	11.8	58.1	93	78.8	25	21.2	
pT classification							
pT1-2	60	29.6	58	96.7	2	3.3	0.002
pT3-4	143	70.4	113	79.0	30	21.0	
pN classification							
pN0	113	55.7	106	93.8	7	6.2	0.0001
pN1-2	90	44.3	65	72.2	25	27.8	
TNM stage							
Stage I	48	23.6	46	95.8	2	4.2	0.0001
Stage II	65	32.0	60	92.3	5	7.7	
Stage III	90	44.3	65	72.2	25	27.8	
pCRM status							
pCRM(−)	189	93.1	164	86.8	25	13.2	0.0002
pCRM(+)	14	6.9	7	50.0	7	50.0	
Tumor grade							
Low-medium	170	83.7	146	85.9	24	14.1	0.144
High	33	16.3	25	75.8	8	24.2	

mrCRM, MRI-assessed circumferential margin; CEA, carcinoembryonic antigen; pCRM, pathological circumferential margin.

**Table 2:** The association between radiological and clinicopathological characteristics and patients' outcomes.

Characteristics	Number (%)	LR			DFS			OS		
		HR	95% CI	*p* value	HR	95% CI	*p* value	HR	95% CI	*p* value
Age, years										
<65	113 (55.7)	1			1			1		
≥65	90 (44.3)	1.40	0.65–3.05	0.393	1.60	0.91–2.83	0.105	3.16	1.38–7.24	0.007
Sex										
Male	104 (51.2)	1			1			1		
Female	99 (48.8)	0.77	0.36–1.69	0.518	0.72	0.41–1.29	0.271	1.06	0.46–2.44	0.899
Preoperative CEA										
<5 ng/*μ*L	147 (72.4)	1			1			1		
≥5 ng/*μ*L	56 (27.6)	2.75	1.27–5.96	0.011	1.11	0.59–2.10	0.749	2.36	1.05–5.34	0.038
mrT classification										
mrT1-2	30 (14.8)	1			1			1		
mrT3	173 (85.2)	30.9	0.59–1606	0.089	4.36	1.35–14.1	0.014	9.01	1.21–67.1	0.032
mrN classification										
mrN (−)	114 (56.2)	1			1			1		
mrN (+)	89 (43.8)	2.12	0.96–4.70	0.063	1.12	0.64–1.98	0.69	1.54	0.69–3.45	0.296
mrCRM										
Clear	171 (84.2)	1			1			1		
Involved	32 (15.8)	4.41	2.05–9.51	0.0001	2.22	1.22–4.05	0.009	3.02	1.30–7.00	0.01
Location of closest mrCRM in bowel wall										
Anterior	107 (52.7)	1			1			1		
Lateral/posterior	96 (47.3)	0.57	0.25–1.28	0.173	0.78	0.44–1.38	0.390	0.75	0.33–1.69	0.483
Peritoneal reflex relationship										
Above	39 (19.2)	1			1			1		
Below	164 (80.8)	2.67	0.80–8.92	0.111	3.07	1.21–7.78	0.018	2.99	0.89–10.0	0.077
Tumor distance from anal verge										
>5 cm	85 (41.9)	1			1			1		
≤5 cm	118 (58.1)	1.56	0.69–3.50	0.284	0.94	0.53–1.65	0.822	1.61	0.69–3.77	0.269
pT classification										
pT1-2	60 (29.6)	1			1			1		
pT3-4	143 (70.4)	12.2	1.65–89.9	0.014	5.43	1.95–15.1	0.001	12.2	1.65–90.7	0.014
pN classification										
pN (−)	113 (55.7)	1			1			1		
pN (+)	90 (44.3)	5.31	2.00–14.1	0.001	2.36	1.31–4.28	0.004	2.67	1.13–6.30	0.025
pCRM										
pCRM (−)	189 (93.1)	1			1			1		
pCRM (+)	14 (6.9)	4.66	1.75–12.4	0.002	2.60	1.10–6.14	0.029	0.92	0.12–6.83	0.933
Tumor grade										
Low-medium	170 (83.7)	1			1			1		
High	33 (16.3)	1.28	0.48–3.42	0.612	1.94	1.01–3.73	0.048	1.41	0.48–4.23	0.536
Adjuvant treatment										
No treatment	67 (33.0)	1			1			1		
Chemotherapy	69 (34.0)	3.65	1.19–11.2	0.024	3.43	1.45–8.12	0.005	5.06	1.43–17.8	0.012
Chemoradiotherapy	67 (33.0)	2.40	0.74–7.85	0.145	3.53	1.49–8.36	0.004	3.34	0.89–12.7	0.076

LR, local recurrence; DFS, disease-free survival; OS, overall survival; mrCRM: MRI-assessed circumferential margin; pCRM: pathological circumferential margin.
